# Geospatial Modelling and Univariate Analysis of Commensal Rodent-Borne Cestodoses: The Case of Invasive spp. of *Rattus* and Indigenous *Mastomys coucha* From South Africa

**DOI:** 10.3389/fvets.2021.678478

**Published:** 2021-06-11

**Authors:** Rolanda S. Julius, Tsungai A. Zengeya, E. Volker Schwan, Christian T. Chimimba

**Affiliations:** ^1^Department of Zoology and Entomology, Department Science & Technology (DST)- National Research Foundation (NRF) Centre of Excellence for Invasion Biology (CIB), University of Pretoria, Pretoria, South Africa; ^2^Kirstenbosch Research Centre, South African Biodiversity Institute, Cape Town, South Africa; ^3^Department of Veterinary Tropical Diseases, University of Pretoria, Pretoria, South Africa

**Keywords:** ecological niche modelling, *Hymenolepis diminuta*, *Hymenolepis nana*, inermicapsifer madagascariensis, parasites, invasive/indigenous murid rodents, species distribution models, South Africa

## Abstract

Poor socio-economic and unsanitary conditions are conducive to commensal rodent infestations, and these conditions are widespread in South Africa. Cestode species of zoonotic interest are highly prevalent in commensal rodents, such as invasive *Rattus norvegicus, Rattus rattus, Rattus tanezumi*, and indigenous *Mastomys coucha*, and have been frequently recovered from human stool samples. These cestode species have similar transmission dynamics to traditional soil-transmitted helminths (STHs), which ties them to infections associated with poverty and poor sanitation. Univariate analysis was used in the present study to determine the association between rodent-related factors and cestode prevalence, while ecological niche modelling was used to infer the potential distribution of the cestode species in South Africa. Cestode prevalence was found to be associated with older rodents, but it was not significantly associated with sex, and ectoparasite presence. The predicted occurrence for rodent-borne cestodes predominantly coincided with large human settlements, typically associated with significant anthropogenic changes. In addition, cestode parasite occurrence was predicted to include areas both inland and along the coast. This is possibly related to the commensal behaviour of the rodent hosts. The study highlights the rodent-related factors associated with the prevalence of parasites in the host community, as well as the environmental variables associated with parasite infective stages that influence host exposure. The application of geospatial modelling together with univariate analysis to predict and explain rodent-borne parasite prevalence may be useful to inform management strategies for targeted interventions.

## Introduction

In South Africa, many households live in informal settlements (1) where there is improper housing infrastructure, poor basic infrastructure such as potable water, sewerage and storm water drainage, and irregular service delivery such as waste disposal and high unemployment rates (2). These circumstances ultimately lead to poor communities living in unsanitary conditions. Poor socio-economic and environmental conditions favour the presence of commensal rodents such as the brown rat (*Rattus norvegicus*), the black rat (*Rattus rattus*) and the house mouse (*Mus musculus*) (3). In Johannesburg, South Africa, it was found that commensal rat infestations were highest in low-income households, low-cost housing suburbs and informal settlements (4). Commensal rodents have been implicated in the transmission and spread of several zoonotic diseases including rat-bite fever, plague, leptospirosis and hantavirus haemorrhagic fever (5). They transmit these diseases either directly, through bites and scratches (6), indirectly through environmental contamination with urine/faeces, or through their arthropod ectoparasites (7). Studies in several countries around the world including Qatar, Jamaica, Thailand, Italy, and the U.K. (8–12) have showed that commensal rodents harbour gastrointestinal helminths that have zoonotic potential and this has also been found to be the case in South Africa (13–15).

Soil-transmitted helminth (STH) or geohelminth infections are most prevalent in the developing world (16) and closely correlated with poverty, poor sanitary conditions and impoverished health services (17). In addition, high levels of unemployment and low levels of maternal education are also correlated with human intestinal infection prevalence (17). STH infections are especially prominent in children (18) and cases have been documented across South Africa (19). Although STH infections are not usually related to mortality, they contribute to significant morbidity and may impact childhood development and influence susceptibility to HIV/AIDS and tuberculosis (20, 21).

The most prominent STHs are nematodes which include the human whipworm (*Trichuris trichiura*), giant intestinal roundworm, (*Ascaris lumbricoides*) and hookworms (*Necator americanus* and *Ancylostoma duodenale*) (18). The dwarf tapeworm [*Hymenolepis* (syn. *Rodentolepis*) *nana*] however, is also considered an STH (21) as it has similar transmission dynamics involving infective eggs in soil substrate. *Hymenolepis nana* is the most common cestode of humans, especially in young children (22, 23) and it is frequently recovered from human stool samples in South Africa (17, 21, 24–26). Apart from humans, the species is also associated with rodent hosts (22) and has a direct lifecycle—it does not require an intermediate host to complete its life cycle. Humans become directly infected when ingesting the eggs through faecal-contaminated food, water or dust (23) and the species becomes abundant through faecal-oral transmission (27). The faecal-oral route is significant in the transmission of STH infections to humans via poor personal hygiene, faecal contaminated soil and water sources (17) and through geophagia (28)—the deliberate consumption of soil. There are also other zoonotic cestodes of concern such as the rat tapeworms, *Hymenolepis diminuta* and *Inermicapsifer madagascariensis* (24, 27, 29). These cestodes, which cause rare zoonotic infections, have arthropods as intermediate hosts and an indirect lifecycle. The arthropod intermediate hosts ingest the eggs from the faecal-contaminated environment where they then develop into larvae (cysticercoids) and eventually, infect final (or definitive) hosts such as rodents (*Rattus* spp., *Mastomys* spp., *Arvicanthis niloticus*) (23, 30, 31) and humans following ingestion of infected arthropods, where they develop into the strobilar or adult stages (27).

Soil-transmitted helminths are primarily associated with low altitude, coastal areas and tropical and sub-tropical climates (24, 32). In addition, edaphic factors such as pH and soil composition may influence the occurrence of geohelminths (28). Cestode eggs can persist for a long time in the soil, sewage sludge and even treated waste water may be an additional source as standard treatment methods are ineffective (33), suggesting that cestode egg occurrence may be widespread.

For many parasite species, their occurrence data may be spatially biassed or improperly georeferenced because of opportunistic sampling of few host individuals or the occurrence data are aggregated resulting in maps with coarse spatial scales (34). To quantify human risk of infection and highlight priority areas for intervention, STHs have been geospatially modelled for many countries including South Africa (35). The focus of these models however, was primarily on nematodes and did not take into account the distribution of the potential reservoir hosts. In addition, these cestodes are not treated through standard human de-worming programmes which selectively target nematodes (30, 36), and therefore, their presence may be an indicator of a poverty-related infection (36). A parallel study (15) confirmed the prevalence of cestodes of zoonotic interest in invasive, commensal rodents, *Rattus tanezumi, R. norvegicus, R. rattus*., and the indigenous multimammate mouse (*Mastomys coucha*) as reservoir hosts in Gauteng Province, South Africa.

The present study used univariate analysis to determine the rodent intrinsic factors related to cestode prevalence and ecological niche modelling (37) to predict underlying factors (i.e., climatic, environmental variables) associated with the cestode infection status of commensal rodents, *R. norvegicus, R. rattus, R. tanezumi*, and *M. coucha* and humans and map these areas to predict hotspots of occurrence. While cestode infection prevalence and distribution associated with humans in Gauteng Province and the rest of South Africa is unknown, this predictive analysis may allow insights into the spatial dynamics of this neglected disease risk to humans and highlight priority areas for intervention. The present study predicted that cestode prevalence is likely to be influenced by host intrinsic factors that impact host susceptibility. In addition, rodent-borne cestodoses are likely to be aggregated throughout the landscape and not uniformly distributed across South Africa because cestode species infective stages have different environmental and climatic requirements that determine their distribution.

## Materials and Methods

### Study Area

**Figure 1:**
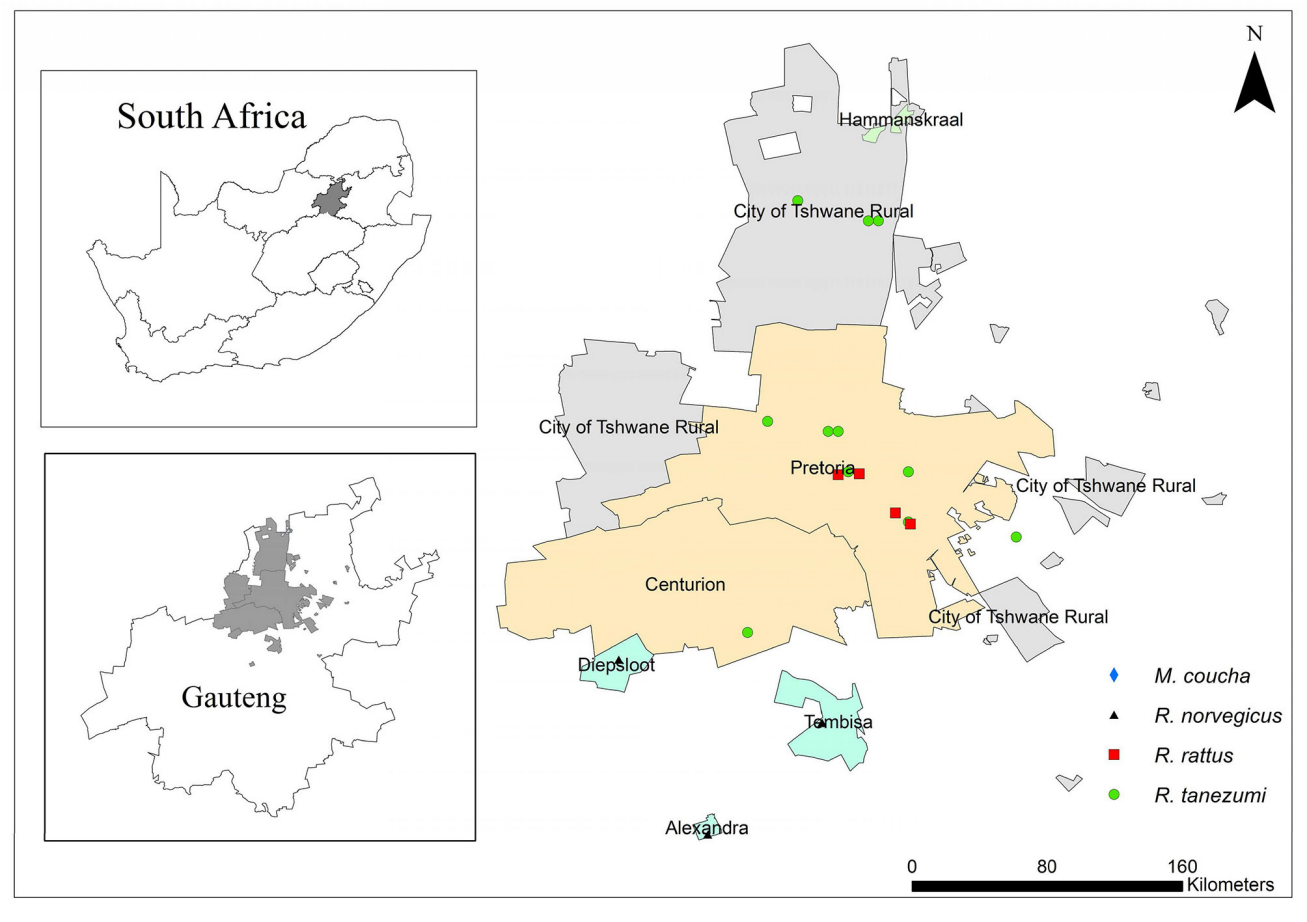
A map of Gauteng Province, South Africa showing sampling localities of commensal, indigenous *Mastomys coucha* and commensal, invasive *Rattus norvegicus, Rattus rattus*, and *Rattus tanezumi*.

Nearly a quarter of the South African population resides in Gauteng Province (38). The province is located inland and it is the smallest of the nine provinces with an area of 18,178 km^2^ that equates to ~1.4% of the total surface area of South Africa (38). This province may therefore be the most densely populated of the nine provinces in the country. On average, the climate is considered temperate but some areas are sub-tropical (39). Trap sites represented urban and peri-urban areas from three of the metropolitan regions namely, the Johannesburg, Ekurhuleni, and Tshwane Metropolitan Municipalities (Figure 1). Geographic coordinates of each trap locality were obtained with a global positioning system (GPS).

### Sample Collection, Rodent, and Cestode Identification

Commensal, invasive *R. norvegicus, R. rattus, R. tanezumi*, and indigenous *M. coucha* species were sampled between August 2010 and September 2011 and in May 2012 using Sherman traps (H.B. Sherman Traps Inc. Florida, USA) and snap traps. Sampling localities were representative of industrial, sub-urban, formal, and informal residential areas as well as smallholdings in Pretoria, Hammanskraal, Diepsloot, Tembisa, and Alexandra (Figure 1). During the 2010–2011 sampling, ~50 snap traps and 100 Sherman live traps were baited with a peanut butter, fish, and oatmeal mixture and placed in and around storage facilities, office buildings and human dwellings. Traps were inspected daily for a trapping period of 1 week per month. In May 2012, *R. norvegicus* samples were also obtained from live-trap captures by the Environmental Health Division of the City of Johannesburg Metropolitan Municipality as part of routine pest control initiatives. All live-trapped rats were transported to the laboratory at the Department of Zoology and Entomology, University of Pretoria, Pretoria while snap-trapped individuals were individually bagged, and transported at 4° C to the same laboratory. All animals sampled in the present study were collected under permit number CPF6 0032 issued by the Gauteng Directorate of Nature Conservation and with the permissions from land owners. Live animals were euthanized by means of halothane inhalation and all carcasses were stored at −20°C until necropsy. Standard measurements were recorded and ectoparasites were collected and stored in absolute ethanol. All procedures on rodents were as approved by the Animal Ethics Committee of the University of Pretoria under ethics clearance number EC025-10.

Rodent identification was based on morphological criteria and molecular techniques. Adult *R. norvegicus* specimens, which have a relatively large body size, were identified based on morphological features such as the tail length in relation to the body length, and the size of the ears relative to the size of the head (40). Adult *R. rattus, R. tanezumi, M. coucha*, and juveniles of all species were identified using mitochondrial cytochrome *b* (cyt *b*) primer sets (41) in conventional polymerase chain reaction (PCR) and genetic sequence analyses following the protocol as described by Julius (42) and Le Grange (43). Cestodes were recovered from the gastrointestinal tract (GIT) of the rodents and stored in glycerol alcohol until identification proceeded based on morphological and molecular criteria (15). Genetic sequences were deposited in the NCBI GenBank database under accession numbers KY462775–9. Morphological identification proceeded with the aid of published taxonomic keys (44, 45) supplemented with species descriptions (13, 46). In many instances, only strobilar fragments without scoleces or strobilar fragments that were too deteriorated to make a conclusive identification of the species were recovered, and therefore individual species abundance could not be calculated. These specimens were subject to molecular identification by PCR and genetic sequence analyses using published genus-specific primer sets (47). Despite these efforts, some specimens could only be identified as cestodes and not to the species level.

A total of 51 occurrence records were obtained of which 16 were generated in this study and 35 were from the literature. Occurrence records for unidentified cestodes, *H. diminuta, H. nana*, and *I. madagascariensis* were obtained from infection status of commensal rodents from Gauteng Province, which was augmented with georeferenced infection status from rodents and humans from other areas in Africa (Supplementary Table 1). These additional georeferenced records were derived from a literature review (Supplementary Table 1), and where exact geographic coordinates were not evident from the publications, these were georeferenced from the study area using Google Maps (48).

### Drivers of Cestode Infection Prevalence Associated With Rodents

**Figure 2:**
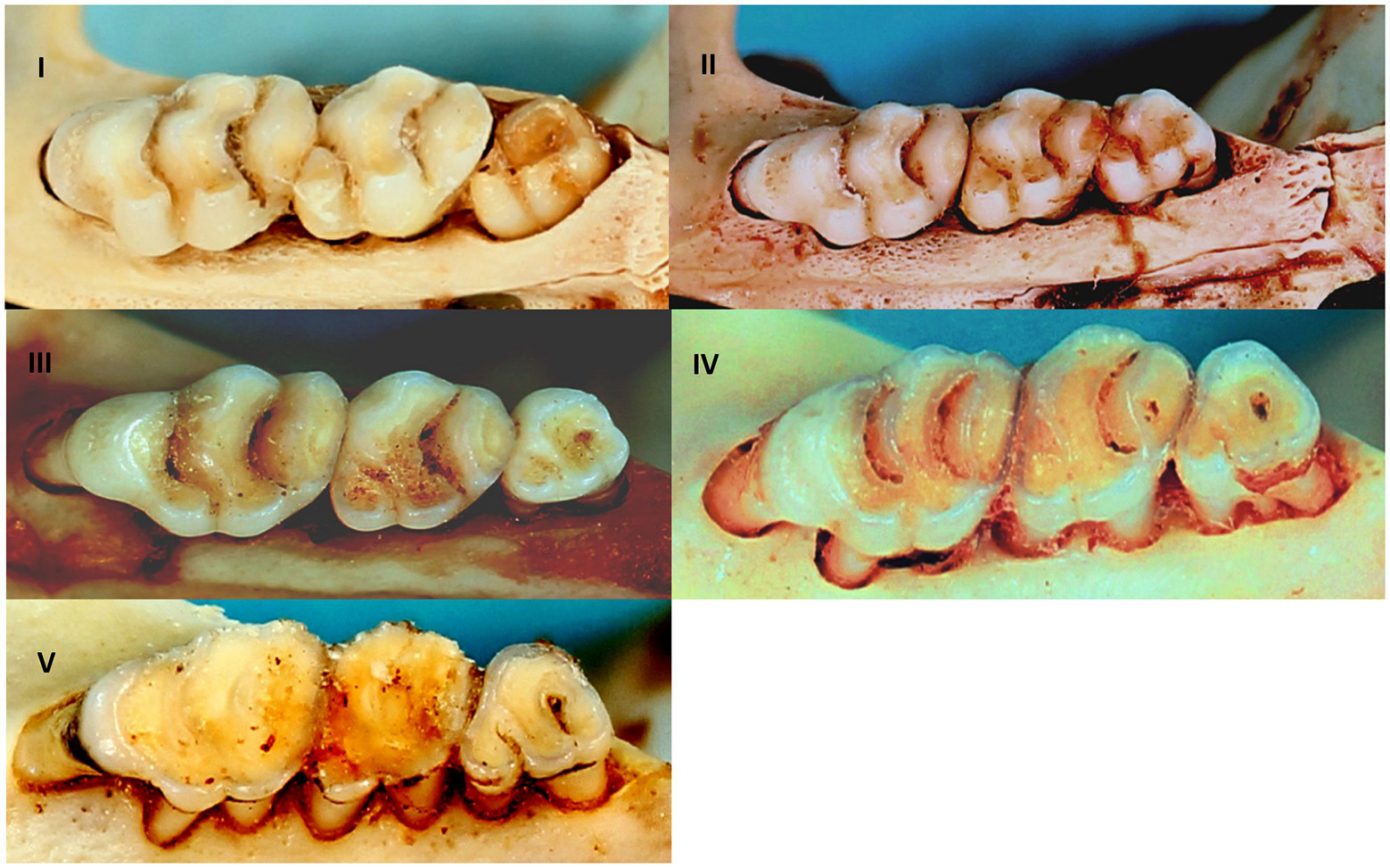
Relative age classes I–V in *R. norvegicus* representing relative age classes in invasive and indigenous commensal, murid rodents from Gauteng Province, South Africa based on the degree of tooth wear of the upper molar tooth row.

It is generally expected that males are more often infected with parasites than females as a result of their behaviour and the negative influence of male testosterone on immune competence (8, 49). This may, however, only apply to the adult cohort for which the sex hormone would be more active (8), and hence there is a need to correct for age. Rodents were sexed morphologically and aged by an assessment of the degree of tooth wear on the upper molar tooth row (50, 51) that led to the assignment of rodent individuals into five relative age classes (Figure 2) for a representative molar row tooth wear pattern as exemplified by *R. norvegicus*. Generalised linear modelling (GLM) applied to the five age classes (I-V) were found to be statistically significant (*P* < 0.05) for nine out of 13 cranial measurements (52). Tukey's *post hoc* analyses indicated some overlap between age classes II & III, and between age classes III & IV (52) and therefore age classes IV–V was considered to be the adult cohort, age classes II–III as the subadult cohort, and age class I as the juvenile cohort. The subadult and adult cohorts were subsequently tested for potential gender effects on cestode prevalence. Ectoparasite identification was beyond the scope of the present study and only the presence or absence was assessed in relation to their intermediate host potential for cestodes that require arthropod intermediate hosts. The association of infection prevalence and seasonality could not be assessed due to the opportunistic sampling of rodents. The chi-square (χ^2^) test, performed in Minitab® v.14 (Minitab Inc., Pennsylvania, U.S.A.), was used to assess the cestode infection prevalence against rodent age, sex and ectoparasite presence.

### Environmental and Climatic Variables Influencing the Occurrence of Cestode Eggs

Environmental variables (Table 1) were selected based on their influence on the survival and establishment of cestode eggs as evident from the literature (53, 54) and which vary within a spatial scale (55). Environmental variables not exclusively associated with cestodes, but associated with STHs predominantly including nematodes on the African continent were also included (28, 56, 57). Soil properties were selected at soil standard depths of 0-30 cm which corresponds to the top soil containing most organic activity. Soil content and property layers were obtained from World Soil Information (58). Remote-sensed data for land surface temperature (LST) and normalised difference vegetation index (NDVI) were extracted from MODIS Terra Satellite (NASA Earth Observing System) using MODIS Toolbox in ArcMap (ESRI Inc., Redlands CA, U.S.A.), while elevation data (DEM) were obtained from African Water Resources Database (AWRD) (59).

**Table 1:** A list of environmental variables used to build ecological niche models of rodent-borne cestodes in Gauteng Province, South Africa.

Clsd1m = Predicted mean clay content at standard soil depth 1 (0–5 cm)
Clsd2m = Predicted mean clay content at standard soil depth 2 (5–15 cm)
Clsd3m = Predicted mean clay content at standard soil depth 3 (15–30 cm)
Sndsd1m = Predicted mean sand content at standard soil depth 1 (0–5 cm)
Sndsd2m = Predicted mean sand content at standard soil depth 2 (5–15 cm)
Sndsd3m = Predicted mean sand content at standard soil depth 3 (15–30 cm)
Sltsd1m = Predicted mean silt content at standard soil depth 1 (0-5 cm)
Sltsd2m = Predicted mean silt content at standard soil depth 2 (5–15 cm)
Sltsd3m = Predicted mean silt content at standard soil depth 3 (15–30 cm)
pHihoxsl1 = Predicted mean pH index (H_2_O solution) at standard soil depth 1 (0–5 cm)
pHihoxsl2 = Predicted mean pH index (H_2_O solution) at standard soil depth 2 (5–15 cm)
pHihoxsl3 = Predicted mean pH index (H_2_O solution) at standard soil depth 3 (15–30 cm)
Orcdrcsl1 = Soil organic carbon at standard soil depth 1 (0–5 cm)
Orcdrcsl2 = Soil organic carbon at standard soil depth 2 (5–15 cm)
Orcdrcsl3 = Soil organic carbon at standard soil depth 3 (15–30 cm)
LST = Land surface temperature
NDVI = Normalised difference vegetation index
DEM = Digital elevation model/altitude [in metres above sea level (a.s.l.)]
GRUMP = Global rural-urban extent
HHI = Human influence index/human footprint
Afripop = African human population size

The urban extent grid (GRUMP) was obtained from the Global Rural-Urban Mapping Project [GRUMP v. 1; Center for International Earth Science Information Network] et al. (60), the human influence index (HHI) or human footprint (Last of the Wild Project v. 2) was obtained from the Wildlife Conservation Society (WCS) and Center for International Earth Science Information Network (61), and the human population density for Africa was obtained from Afripop (62). Data related to temperature and precipitation were extracted from WorldClim database (63, 64) (Table 2), and these bioclimatic variables are widely used in ecological niche modelling of species (63).

**Table 2:** A list of bioclimatic variables used to build ecological niche models of rodent-borne cestodes in Gauteng Province, South Africa.

BIO1 = Annual mean temperature
BIO2 = Mean diurnal range [Mean of monthly (max temp–min temp)]
BIO3 = Isothermality (P2/P7) (* 100)
BIO4 = Temperature seasonality (standard deviation *100)
BIO5 = Max temperature of warmest month
BIO6 = Min temperature of coldest month
BIO7 = Temperature annual range (P5–P6)
BIO8 = Mean temperature of wettest quarter
BIO9 = Mean temperature of driest quarter
BIO10 = Mean temperature of warmest quarter
BIO11 = Mean temperature of coldest quarter
BIO12 = Annual precipitation
BIO13 = Precipitation of wettest month
BIO14 = Precipitation of driest month
BIO15 = Precipitation seasonality (coefficient of variation)
BIO16 = Precipitation of wettest quarter
BIO17 = Precipitation of driest quarter
BIO18 = Precipitation of warmest quarter
BIO19 = Precipitation of coldest quarter

### Ecological Niche Modelling and Distribution of Cestode Species in Gauteng Province, South Africa

Modelling of the distribution of cestode species in Gauteng Province, South Africa was conducted using the maximum entropy approach in MaxEnt v.3.4.1 (37) to identify areas which are potentially suitable for the occurrence of infective stages of selected cestode species that are responsible for rodent-borne cestodoses in Gauteng Province, South Africa. For all models, the algorithm's parameters were set to a maximum number of 500 iterations, a regularisation multiplier of 1, convergence threshold of 0.00001, test percentage of 50, and only hinge features selected (65, 66). In addition, clamping was selected to minimise predictions to regions of environmental space outside the limits encountered during training because extrapolation may over-inflate the degree to which species niches are estimated to overlap (67–69). The logistic output format was used to indicate the probability of a species presence at a default prevalence of 0.5 (66, 70). Values range from 0, indicating low probability, to 1.0, indicating greatest probability of a species presence in a given area.

The predictive ability of ecological niche models is strongly influenced by the selection of variables used to train the models. Various procedures are available to pre-select covariates (71). The present study utilised the in-built method of regularisation in MaxEnt that deals with the selection of environmental variables (regulating some to zero). This approach has been shown to perform well and is considered to out-perform other pre-selection procedures (66, 70). The correlations among variables were tested in the SDM Toolbox [(72); www.sdmtoolbox.org] in ArcMap 10.4 [Environmental Systems Research Institute (ESRI) Inc., Redlands CA, U.S.A.], and for correlated variables, only the more biologically informative variables were retained.

### Background Selection

MaxEnt uses presence and pseudo-absences or background localities to project potential species distribution models (37). The extent of the background is known to influence model performance, where a broad background can cause over-estimates and a constrained background can cause under-estimates (73, 74). In the present study, the background extent for each cestode species was limited to areas that had similar climates to each species' occurrence records in Africa. This was achieved by overlaying the recent Köppen-Geiger climate classification system (75) with the occurrence records for each species following Thompson et al. (76). The Köppen-Geiger polygons identify areas with similar climates (climate zones) and a given climate zone was included as part of the background if it contained an occurrence record within the respective range of each cestode species, using ArcGIS® v. 10.4 (ESRI, Redlands CA, U.S.A.). By selecting the entire climatic zone, an intermediate background size was obtained, compensating for the few occurrence records obtained and capturing the expected distribution of each cestode species (73, 77). For each species, models were calibrated with 10,000 pseudo-absence points drawn at random from the species defined background (37, 65). Occurrence records were randomly partitioned using a *K*-fold approach into equal sets (50%) for calibration and validation (37). Ten niche models were then constructed for each cestode species and a consensus map was then created as an average of the 10 iterations for each species. The predictions for each species were then projected to South Africa.

### Ecological Niche Model Performance Evaluation

The performance of ecological niche models of each cestode species were evaluated using the Maximum test Area Under the Curve (AUC) (78). AUC defines the discrimination ability (between presence and background) of the models where values range from 0 (indicating random distribution) to 1.0 (indicating perfect prediction), with values >0.5 being considered to indicate that the model discriminates better than random (78). All AUC model performance measures were calculated in MaxEnt and predictions with an AUC value were considered to be average if >0.7, good at >0.8 and excellent at >0.9 (79, 80).

## Results

### Univariate Analyses of Cestode Prevalence in the Rodent Community

A total of 395 rodents was examined and comprised *M. coucha* (*n* = 84), *R. norvegicus* (*n* = 240), *R. rattus* (*n* = 40), and *R. tanezumi* (*n* = 31). Cestode infection prevalence among rodent species was reported in Julius et al. (15) as 11% in *M. coucha*, 33% in *R. norvegicus*, 43% in *R. rattus*, and 48% in *R. tanezumi*.

The analysis for cestode infection prevalence between the sexes was restricted to the sub-adult (age classes II-III) and adult cohorts (age classes IV–V; see methods section for justification) and it showed no statistically significant difference between male and female rodent hosts (χ^2^ = 0.60; *df* = 1; *n* = 340; *P* > 0.05). There was a statistically significant difference however, in infection prevalence between each age cohort of the rodent hosts (χ^2^ = 39.36; df = 2; *n* = 395; *P* < 0.05) with infection prevalence increasing from juvenile (age class I; 0.83%, *n* = 120), to sub adult (age classes II–III; 29.17%, *n* = 120) and adult (age classes IV–V; 70%, *n* = 120) rodents. Cestode infection prevalence was not associated with ectoparasite presence (χ^2^ = 0.056; *df* = 1; *n* = 395; *P* > 0.05).

### Potential Distribution of Rodent-Borne Cestodes

**Figure 3:**
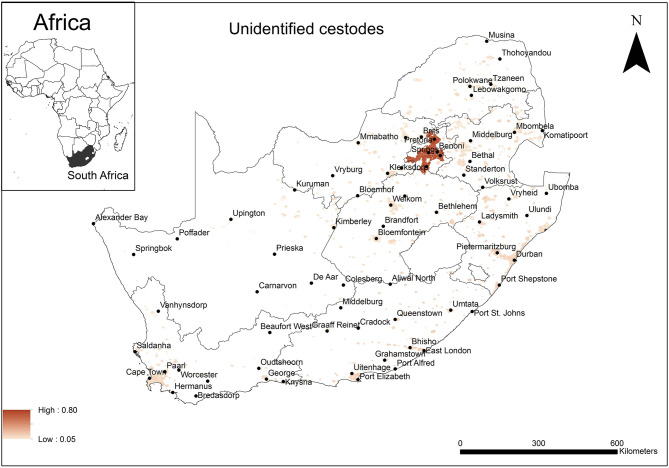
A map showing predicted occurrence of rodent-borne cestodoses in South Africa based on environmental suitability (shaded areas). White (non-shaded) areas represent areas where data were unavailable.

The areas that were predicted as suitable (probability of presence > 0.5) for unidentified rodent-borne cestode species occurrence were associated mainly with urban areas in Gauteng Province (Figure 3). Model performance was good (AUC = 0.81) and the variable that contributed most to model performance was the global rural-urban extent (94.9%) areas predicted as highly suitable for the establishment of cestodes had large urban extent (range 7,500–8,000 km^2^, optimum = 8,000 km^2^).

**Figure 4:**
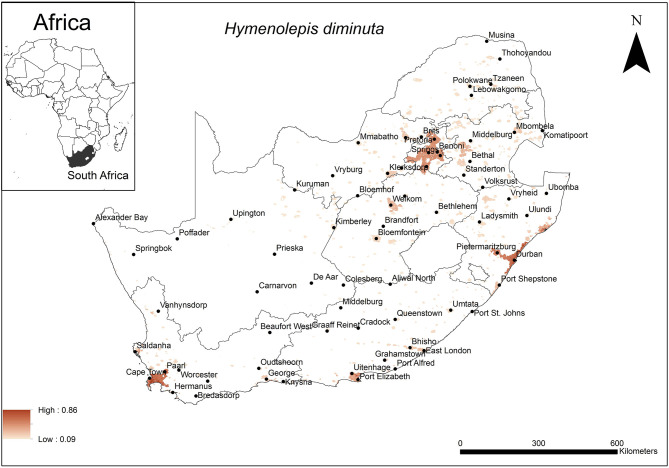
A map showing predicted occurrence of *H. diminuta* in South Africa based on environmental suitability (shaded areas). White (non-shaded) areas represent areas where data were unavailable.

The areas were predicted to be highly suitable for *H. diminuta* occurrence were associated with major urban areas along the coast and the inland Gauteng Province of South Africa (Figure 4). These included the metropolitan areas around the cities of Cape Town, Port Elizabeth and Durban and certain areas in Gauteng Province. The model performance was good (AUC = 0.80) and the variables that contributed most to model performance were global rural-urban extent (65.6%) and altitude (28.8%). The areas that were predicted as highly suitable were mainly located in areas which had a large urban extent (range = 2,000–7,837 km^2^, optimum = 7,837 km^2^) and low altitude (range = 0–1,200 m a.s.l., optimum = 0 m a.s.l.).

**Figure 5:**
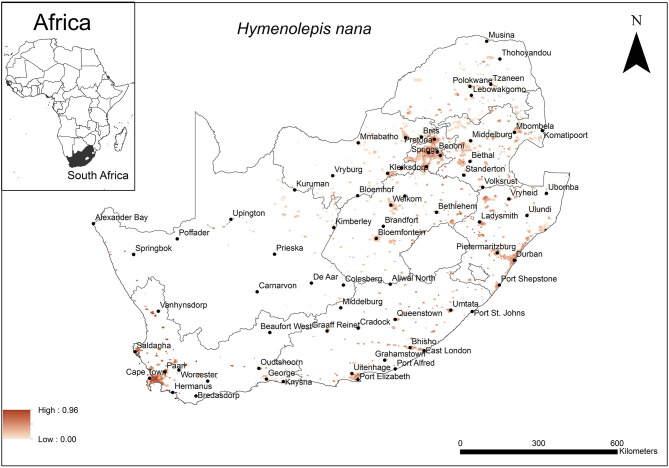
A map showing predicted occurrence of *H. nana* in South Africa based on environmental suitability (shaded areas). White (non-shaded) areas represent areas where data were unavailable.

The areas that were predicted as highly suitable for *H. nana* occurrence were widespread across South Africa (Figure 5). These include cities along the coast (Cape Town, Durban, East London, Port Elizabeth) as well as inland metropolitan areas such as Pietermaritzburg, Bloemfontein, Pretoria and Johannesburg.

The model performance was average (AUC = 0.79) and the variables that contributed most to model performance were precipitation of the warmest quarter (29.4%), human footprint (27.2%), and soil silt fraction at soil depth of 15–30 cm (15.3%). The areas which were predicted as highly suitable were mainly associated with areas which received an average precipitation of 50 mm (range = 50–100 mm) in the warmest quarter, a high relative anthropogenic impact (range = 45–65%, optimum = 65%) and relatively low silt content in the lowest layer of the topsoil (depth 15–30 cm) (range = 16–18%, optimum = 18%).

**Figure 6:**
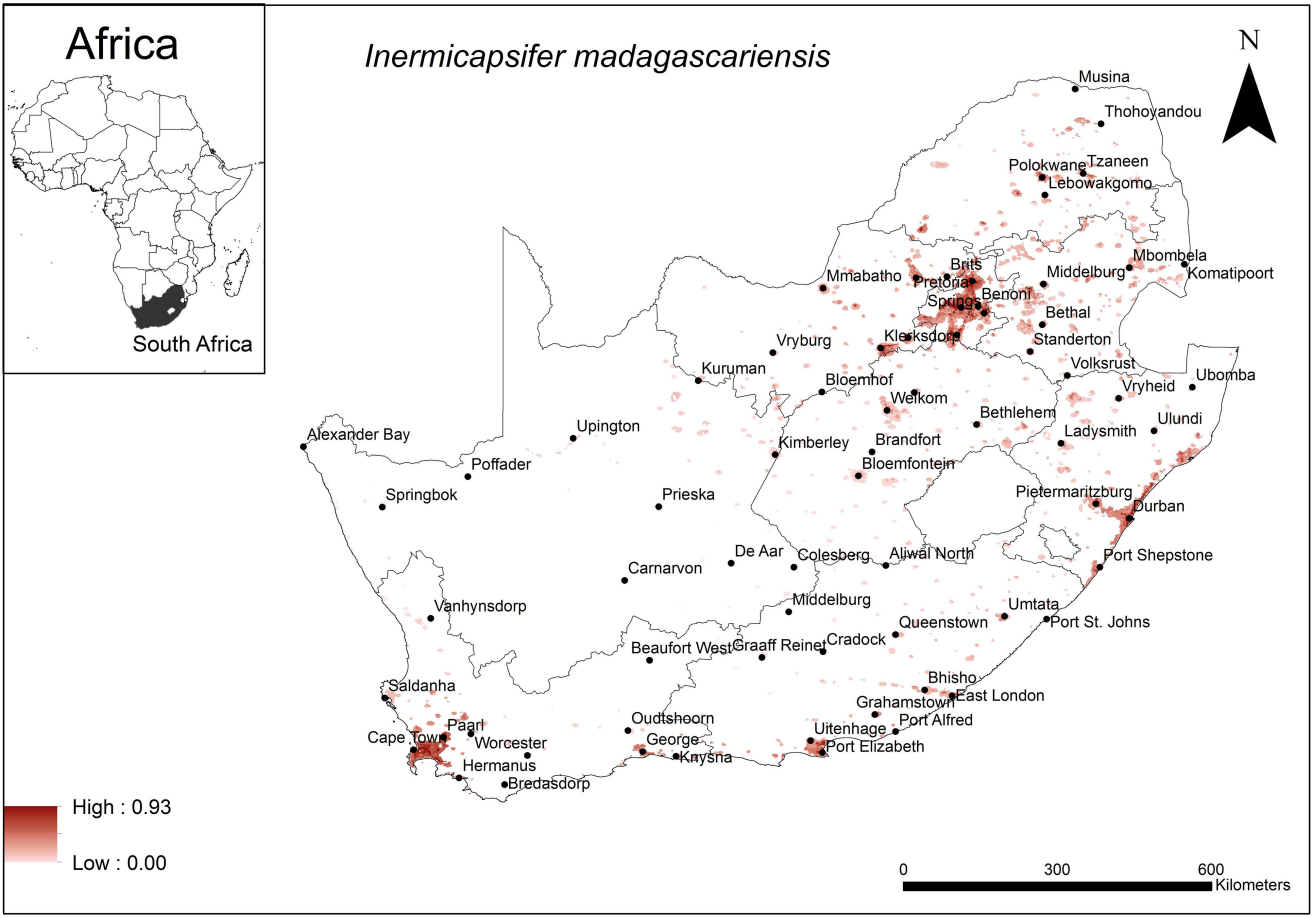
A map showing predicted occurrence of *I. madagascariensis* in South Africa based on environmental suitability (shaded areas). White (non-shaded) areas represent areas where data were unavailable.

The areas that were predicted as suitable for *I. madagascariensis* occurrence were associated with major urban areas in South Africa (Figure 6). These included the large cities of Cape Town, Durban, Port Elizabeth, Pretoria and Johannesburg. Model performance was average (AUC = 0.72) and the variables that contributed most to model performance were human footprint (37.1%), annual precipitation (28%) and global rural-urban extent (11.4%). The areas which were predicted as highly suitable were mainly associated with high human footprint (range = 42–62%, optimum = 62%), high annual precipitation (range = 600–800 mm, optimum = 800 mm) and large urban extent (range = 500–12,593 km^2^, optimum = 12,593 km^2^).

**Figure 7:**
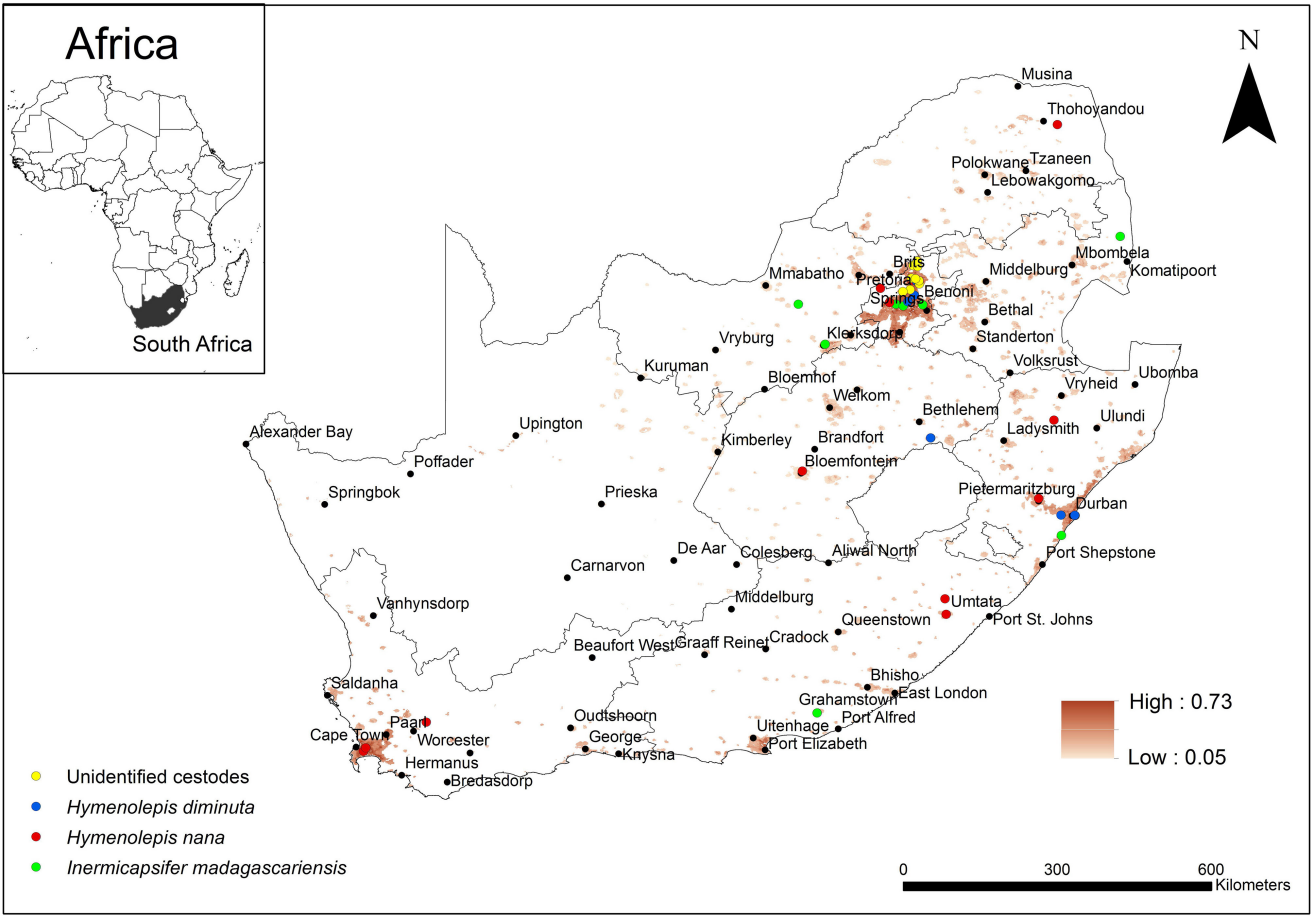
Map showing the predicted occurrence of rodent-borne cestode taxa (weighted 25%) in South Africa based on environmental suitability (shaded areas) along with known occurrence records represented by yellow, blue, red, and green circles. White (non-shaded) areas represent areas where data were unavailable.

A summarised projection of all cestode taxa was produced by equally weighting (25%) each of the taxa (Figure 7). The predicted occurrence of *H. diminuta* overlapped with areas of known distribution in the KwaZulu-Natal, Free State, and Gauteng Provinces but in addition it was predicted to also occur in the Eastern Cape and Western Cape Provinces. *Hymenolepis nana* was predicted to occur in all the areas of known distribution which included Limpopo, Gauteng, Free State, Western Cape, Eastern Cape, and KwaZulu-Natal Provinces but in addition, it was also predicted to occur in the Northern Cape, North West and Mpumalanga Provinces. *Inermicapsifer madagascariensis* has a known distribution in Mpumalanga, Gauteng, North West, KwaZulu-Natal, and Eastern Cape Provinces which coincided with its predicted distribution, but in addition, it was also predicted to occur in the Western Cape and Limpopo Provinces.

## Discussion

This study assessed factors that could affect the prevalence of cestodes in commensal rodents such as *R. norvegicus, R. rattus, R. tanezumi*, and *M. coucha* and applied ecological niche modelling to identify climatic and environmental variables associated with cestode infection of the rodents and to predict areas were the cestodes could occur. Cestode prevalence was found to be associated with the age of the rodents but other factors such sex, and presence or absence of ectoparasites were not significant. The predicted occurrence for all the assessed rodent-borne cestodes predominantly coincided with large human settlements, but there were some species-specific differences in factors that influenced potential distribution that could be related to the ecology of each species.

### Rodent-Related Variables and Cestode Prevalence

Host variability in parasite burden should influence the establishment and spread of parasites (49). As predicted, cestode prevalence was shown to be associated with at least one of the three host intrinsic factors tested namely, sex, age, and ectoparasite prevalence. The prediction that cestode infection prevalence should be biassed toward male rodent hosts was not observed and there was no sex effect observed for cestode infection in the adult cohort. In addition to age structure however, seasonality may also influence sex-biassed parasite prevalence as is the case in certain parasites of voles when the female cohort survived the overwintering period better than the male cohort (49) resulting in a seasonal sex bias for that parasite. Due to the opportunistic sampling of the rodent hosts, this aspect could not be assessed. Rodent age, however, had a significant effect on cestode prevalence where older rodents had higher infection prevalence than younger rodents. This trend is typically observed when parasite transmission rates in the host population are low, allowing decreased parasite loads in younger individuals that result in slow acquired immunity and this in turn leads to the parasite infection to peak with increased age (49). Transmission rates may be low because the rodent species are commensal and may therefore feed more on food items found in and around human dwellings than on natural food items such arthropods, which are potential intermediate hosts of cestode species. The lack of association between ectoparasite prevalence and cestode infection was somewhat surprising as one would expect transmission to be enhanced through grooming activities that led to ingestion of infected arthropods. *Hymenolepis diminuta* has a wide range of arthropod intermediate hosts including fleas, beetles, and moths (49). The ectoparasites observed on invasive *Rattus* species primarily included lice while for the indigenous *M. coucha* these were rodent-specific mites (R. Julius pers. obs.). The taxonomic status of the ectoparasites taxa may therefore not have been the target arthropod taxa involved in the life cycle of these cestode taxa. Another intrinsic factor that may influence parasite prevalence which was not assessed in the present study are co-parasitic interactions (either direct or host immune mediated) (49). It is suggested however, that if intrinsic factors of host sex, age, and reproductive status have been accounted for, interspecific interactions among parasites may be negligible (49).

### Environmental Variables

The heterogeneous environment influences the host exposure to infective stages of the parasite (49). It is evident that from the over 40 environmental variables used in the model, only a select few contributed to model performance. This suggests that a few variables may be limiting the potential distribution of the cestode species. These environmental variables associated with cestode infective stages were shown to be good predictors of rodent-borne zoonotic cestodoses and supports the prediction that rodent-borne cestodoses are not uniformly distributed across the South African landscape.

The unidentified rodent-borne cestodes are predominantly associated with the size of the urban settlement reaching an optimal size at 8,000 km^2^. The urban extent takes into account the amount of night-time lights reflected, known urban settlements and human population size (81). The predicted distribution is concentrated in Gauteng Province, which contains two of the largest cities namely Johannesburg and Pretoria, and when combined, the two cities constitute the most populated urban area in South Africa (38). Perhaps the human population density, mobility and rodent-human proximity, facilitate transmission, and maintain a continued cestode presence.

The predicted distribution of the rat tapeworm, *H. diminuta* was mainly associated with urban extent and altitude being optimal at 7,837 km^2^ and 0 metres a.s.l., respectively. This coincides with the major cities and large towns at low altitude along the coast. Some areas in Gauteng Province are also included in the predicted distribution but because these areas are situated inland at high altitude, the governing element here is likely to be the size of the urban area. Coincidentally, urban extent, which was also selected to influence the distribution of the unidentified rodent-borne cestodes, has nearly the same optimal size of the urban area which is implicated to account for the extent of occurrence of *H. diminuta*. Similarly, human population, human mobility and the close proximity of rodents may therefore, also facilitate the transmission of this cestode species. The associated low altitude coastal area has previously been recognised as a factor that influence the prevalence of STHs (32, 56) particularly nematodes. The sandy soils associated with coastal areas favour the establishment of the hookworm, *N. americanus* and threadworm, *Strongyloides stercoralis*, as it allows mobility of the free-living larvae to avoid desiccation or submergence by rising water levels (28, 82). Similarly, in the case of *H. diminuta*, the sandy soils likely favour their distribution through mobility of cysticercoid infected arthropods. Walker et al. (32) however, demonstrated that STHs including *H. nana* also occurred in high altitude areas such as Gauteng Province and the current study emphasised this.

Unlike many cestode species, the dwarf tapeworm, *H. nana*, does not require an intermediate host to complete its lifecycle (27) and eggs are passed directly into the environment and ultimately in soil substrate. It is expected that this may be reflected in the environmental variables selected to predict its distribution. The predicted distribution appears to be widespread across South Africa where human settlements occur and where the summer rainfall allows precipitation of at least 50 mm with top soil having a silt component close to 20%. For this cestode species that has adapted to a direct life cycle, climatic factors may be very important in the survival of the eggs. Parasite transmission is favoured in the warmest period of the year and egg survival is associated with moist environments (49), which may explain why precipitation of the summer season and soil composition were selected as the most important variables in model performance. Most areas in South Africa that were predicted suitable for the cestode species receive summer rainfall of at least 50 mm (83). The human footprint index quantified the anthropogenic changes related to the landscape (84). Its selection as one of the important variables in model selection was expected because *H. nana* is associated with commensal, invasive *Rattus* species which occupy human settlements where the anthropogenic changes to the landscape would be expected to be highest. Anthropogenic changes can result in clumped resources which may cause aggregation of hosts which in turn influence parasite transmission (85).

The environmental variables that account for the predicted occurrence of *I. madagascariensis* are human footprint, annual precipitation, and the urban extent which were found to be optimal at 62%, 800 mm and 1,2593 km^2^, respectively. The predicted distribution of this cestode species is widespread but particularly associated with major cities of South Africa which may be explained by the large urban extent and high human footprint selected as optimal criteria. In this case, the human population, mobility, close human contact with commensal indigenous *M. coucha* and associated anthropogenic changes may contribute in the survival and transmission of the cestode. Soil-dwelling oribatid mites are believed to be intermediate hosts of *I. madagascariensis* (30) and annual precipitation may influence the occurrence of mites as they emerge to the soil surface upon irrigation (86). Annual precipitation at a minimum of 600 mm occurs widely in South Africa with the exception of the Northern Cape Province (87) and is indicated for some but not all of the areas of predicted distribution for this species. Annual precipitation is therefore not likely to be a limiting factor.

Some areas predicted as suitable for rodent-borne cestodes included areas that had no prior record of occurrence for the cestode species. This highlights that the species is likely to be under-sampled. Under-estimation of helminth infections is common due to challenges associated with occult infections and lack of mandatory reporting in most countries (54). The present study therefore, highlights the current and potential areas at risk of the establishment of rodent-borne cestodes and identifies the areas that require data collection through surveys.

## Conclusion

Rodent age as an intrinsic factor was found to impact on strobilar cestode prevalence in the rodent hosts and supports the prediction that cestode prevalence is aggregated among the rodent community which is biassed to the older rodents in the present study. Comprehensively sampled data from surveys that are correctly georeferenced will remain the ideal method to map species distributions. In disease ecology and epidemiology, however, this will rarely be the case as the urgency to allocate resources and implement interventions is of primary importance following reported cases. Geospatial models are valuable as they not only identify the areas at risk but also the factors which may influence the occurrence. In the present study the modelling approach produced statistically supported SDMs of cestode species at a coarse resolution and yet it could be interpreted in context of the niche requirements of the species. Urban extent and human footprint were implicated in the predicted distribution of more than one of the cestode species assessed in this study and favour the commensal nature of the definitive rodent hosts. In addition, altitude and soil characteristics that may impact the arthropod intermediate hosts were also found as predictors for potential distribution. The study determined that the ecological niche of the cestode species were influenced by host intrinsic factors and environmental factors that impact infective stages not only directly but also indirectly through the environment of the intermediate and definitive hosts. Management interventions to combat disease emergence need to take into account all such factors to have any chance of success. The combination of geospatial modelling and univariate analyses could allow management plans to rapidly identify critical areas and vectors to implement a targeted response.

## Data Availability Statement

The genetic sequence data presented in the study are deposited in the NCBI GenBank repository, accession number KY462775–9.

## Ethics Statement

The animal study was reviewed and approved by Animal Ethics Committee of the University of Pretoria.

## Author Contributions

RJ performed sample collection, data analyses, and wrote the manuscript. TZ and ES performed data analyses, revised, and edited the manuscript. CC reviewed and edited the manuscript. All authors contributed to the article and approved the submitted version.

## Conflict of Interest

The authors declare that the research was conducted in the absence of any commercial or financial relationships that could be construed as a potential conflict of interest.
