# Supplementary material for: Geospatial Modelling and Univariate Analysis of Commensal Rodent-Borne Cestodoses: The Case of Invasive spp. of Rattus and Indigenous Mastomys coucha From South Africa
Source: Front Vet Sci. 2021 Jun 11;8:678478. doi: 10.3389/fvets.2021.678478 (PMC8226005; doi:10.3389/fvets.2021.678478)
Supplement: Supplementary file 1 [file Table_1.DOCX]

**Supplementary material**

**Table S1.** Occurrence records for *Inermicapsifer madagascariensis*, *Hymenolepis diminuta*, *Hymenolepis nana* used in the species distribution models of rodent-borne cestode species in South Africa

| **Reference** | **Cestode species** | **Host** | **Geographic locality** | | | **GPS coordinates** | | **Source*** |
| --- | --- | --- | --- | --- | --- | --- | --- | --- |
| Collins 1972 | *I. madagascariensis* | *R rattus* | South Africa | Gauteng | Pretoria | -25.74 | 28.17 | inferred |
| Collins 1972 | *I. madagascariensis* | *M natalensis* | South Africa | Mpumalanga | Kruger National Park | -24.97 | 31.82 | inferred |
| Sall-Drame e tal. 2010 | *I.madagascariensis* | *Arvicanthis niloticus* | Senegal | Lake Retba |  | 14.83 | 17.23 | published |
| Brouat et al. 2007 | *I.madagascariensis* | *M natalensis* | Senegal | Kedougou |  | 12.56 | 12.17 | published |
| Brouat et al. 2007 | *H. nana* | *Mastomys erythroleucus* | Senegal | Kedougou |  | 12.56 | 12.17 | published |
| Goldsmid 1972 | *I. madagascariensis* | *M natalensis* | Zimbabwe |  | Harare | -17.88 | 31.02 | inferred |
| Archer et al. 2017 | *H. diminuta* | *R norvegicus* | South Africa | KwaZulu Natal | Durban | -29.86 | 31.03 | inferred |
| Archer et al. 2017 | *H. nana* | *R norvegicus* | South Africa | KwaZulu Natal | Durban | -29.86 | 31.03 | inferred |
| Archer et al. 2017 | *H. diminuta* | *R rattus* | South Africa | KwaZulu Natal | Dassenhoek | -29.85 | 30.78 | inferred |
| Mafiana et al. 1997 | *H. diminuta* | *R rattus* | Nigeria |  | Abeokuta | 12.56 | -12.17 | inferred |
| Diagne et al. 2016 | *H. diminuta* | *R rattus* | Senegal | Kedougou |  | 13.81 | -12.72 | inferred |
| Diagne et al. 2016 | *H. diminuta* | *R rattus* | Senegal | Soutouta |  | -26.11 | 28.10 | inferred |
| this study | *H. diminuta* | *R. norvegicus* | South Africa | Gauteng | Alexandra | -26.11 | 28.10 |  |
| this study | *H. nana* | *R. norvegicus* | South Africa | Gauteng | Alexandra | -25.48 | 28.19 |  |
| this study | cestode | *R tanezumi* | South Africa | Gauteng | Hammanskraal | -25.50 | 28.27 |  |
| this study | cestode | *R tanezumi* | South Africa | Gauteng | Hammanskraal | -25.40 | 28.26 |  |
| this study | *I. madagascariensis* | *M coucha* | South Africa | Gauteng | Hammanskraal | -25.40 | 28.26 |  |
| this study | cestode | *R tanezumi* | South Africa | Gauteng | Hammanskraal | -25.91 | 28.14 |  |
| this study | cestode | *R tanezumi* | South Africa | Gauteng | Pretoria | -25.94 | 28.01 |  |
| this study | cestode | *R norvegicus* | South Africa | Gauteng | Diepsloot | -25.80 | 28.30 |  |
| this study | cestode | *R tanezumi* | South Africa | Gauteng | Pretoria | -25.75 | 28.25 |  |
| this study | *H. diminuta* | *R rattus* | South Africa | Gauteng | Pretoria | -25.75 | 28.25 |  |
| this study | *I. madagascariensis* | *M coucha* | South Africa | Gauteng | Pretoria | -25.70 | 28.16 |  |
| this study | *cestode* | *R tanezumi* | South Africa | Gauteng | Pretoria | -26.00 | 28.21 |  |
| this study | *H. diminuta* | *R norvegicus* | South Africa | Gauteng | Tembisa | -25.75 | 28.30 |  |
| this study | cestode | *R tanezumi* | South Africa | Gauteng | Pretoria | -25.71 | 28.23 |  |
| this study | cestode | *R tanezumi* | South Africa | Gauteng | Pretoria | 25.37 | 28.28 |  |
| this study | *I. madagascariensis* | *M coucha* | South Africa | Gauteng | Hammanskraal | -17.88 | 31.02 |  |
| Adeleke et al. 2015 | *H. nana* | human prevalence | South Africa | Eastern Cape | Mthatha General Hospital | -31.59 | 28.77 | inferred |
| Gumbo et al. 2010 | *H. nana* | human prevalence | South Africa | Limpopo | Malamulele | -23.01 | 30.72 | published |
| Collins 1972 | *I. madagascariensis* | human prevalence | South Africa | Gauteng | Pretoria | -25.74 | 28.17 | inferred |
| van Niekerk et al. 1979 | *H. nana* | human prevalence | South Africa | Western Cape | Gugulethu | -33.99 | 18.56 | inferred |
| van Niekerk et al. 1979 | *H. nana* | human prevalence | South Africa | Eastern Cape | Tsolo | -31.32 | 28.75 | inferred |
| Kark & Le Riche 1944 | *H. nana* | human prevalence | South Africa | Free State | Bloemfontein | -29.08 | 26.25 | inferred |
| Kark & Le Riche 1944 | *H. nana* | human prevalence | South Africa | Gauteng | Pretoria | -25.74 | 28.17 | inferred |
| Kark & Le Riche 1944 | *H. nana* | human prevalence | South Africa | KwaZulu Natal | Nqutu | -28.19 | 30.66 | inferred |
| Kark & Le Riche 1944 | *H. nana* | human prevalence | South Africa | KwaZulu Natal | Pietermaritzburg | -29.56 | 30.39 | inferred |
| Frean & Dini 2004 | *I. madagascariensis* | human prevalence | South Africa | Gauteng | Roodepoort | -26.16 | 27.89 | inferred |
| Frean & Dini 2004 | *I. madagascariensis* | human prevalence | South Africa | North West Province | Klerksdorp | -26.86 | 26.65 | inferred |
| Frean & Dini 2004 | *I. madagascariensis* | human prevalence | South Africa | Gauteng | Benoni | -26.17 | 28.36 | inferred |
| Frean & Dini 2004 | *I. madagascariensis* | human prevalence | South Africa | North West Province | Lichtenburg | -26.16 | 26.18 | inferred |
| Frean & Dini 2004 | *I. madagascariensis* | human prevalence | South Africa | KwaZulu Natal | Ukomaas | -30.21 | 30.79 | inferred |
| Frean & Dini 2004 | *I. madagascariensis* | human prevalence | South Africa | Eastern Cape | Grahamstown | -33.32 | 26.51 | inferred |
| Frean & Dini 2004 | *I. madagascariensis* | human prevalence | South Africa | Gauteng | Johannesburg | -26.19 | 28.02 | inferred |
| Goldsmid 1972 | *I. madagascariensis* | human prevalence | Zimbabwe |  | Harare | -17.84 | 31.02 | inferred |
| Adams et al. 2005 | *H. nana* | human prevalence | South Africa | Western Cape | Parow | -33.93 | 18.60 | inferred |
| Walker 2000 | *H. nana* | human prevalence | South Africa | Gauteng | Kagiso | -26.13 | 27.78 | published |
| Walker 2000 | *H. nana* | human prevalence | South Africa | Gauteng | Hekpoort | -25.88 | 27.62 | published |
| Fincham et al. 1996 | *H. nana* | human prevalence | South Africa | Western Cape | De Doorns | -33.48 | 19.66 | inferred |
| Mosala 1995 | *H. nana* | human prevalence | South Africa | Free State | Qwa-Qwa | 28-30S | 28-30E | published |
| Mosala 1995 | *H. diminuta* | human prevalence | South Africa | Free State | Qwa-Qwa | 28-30S | 28-30E | published |

*Inferred georeferenced coordinates from study area using Google Maps
